# The Baby’s Airing

**Published:** 1893-02

**Authors:** 


### THE BABY'S AIRING.

It is well to send the babies out for an airing every day, if they are confided to competent hands. But often baby's tender little body is jarred and wearied by being rattled over a rough road, bounced into and over gutters, and thumped over crossings at headlong speed, until it receives more harm than good from its outing. Almost every one knows what a difference there is in drivers; how one man will, however easy the carriage, take you to your journey's end feeling that you are black and blue from jolting about while another will avoid every loose stone and moderate his speed at the rough places. Be sure that babies suffer quite as much as their elders from unskillful charioteers. It is perfectly easy to guide a childs cab over a gutter without a jar, but it is seldom done by a servant, and often not by mothers themselves. Not only are the little ones jerked and bumped along in this tiresome fashion, but they are kept hours in their carriages without change of position, getting benumbed and cold in consequence. This is quite wrong. Young infants should take the air in the arms of an attendant. Very serious evils result from subjecting their tender bodies tojars.

